# Confirmatory factor analysis and gender invariance of Persian version of the modified Yale food addiction scale (mPYFAS) 2.0: insight from a large scale Iranian sample

**DOI:** 10.1186/s40337-023-00962-1

**Published:** 2024-01-23

**Authors:** Mohammad Niroumand Sarvandani, Masoud Asadi, Balal Izanloo, Maryam Soleimani, Faezeh Mahdavi, Ashley N. Gearhardt, Qing-Wei Chen, Nasrin Ghadiri Varzaneh, Roya Taghadosiniya, Hamed Ghazvini, Maryam Khoramrooz, Raheleh Rafaiee

**Affiliations:** 1 Addiction Studies Department, School of Medicine, Shahroud University of Medical Sciences, Shahroud, Iran; 2 Department of Psychology, Faculty of Humanities, Arak University, Arak, Iran; 3 Faculty of Psychology and Education, Kharazmi University, Tehran, Iran; 4 Department of Psychology and Counseling, Faculty of Humanities, University of Hormozgan, Bandar Abbas, Iran; 5 The Health of Plant and Livestock Products Research Center, Mazandaran University of Medical Sciences, Sari, Iran; 6 Department of Psychology, University of Michigan, Ann Arbor, MI USA; 7 National Center for International Research on Green Optoelectronics, South China Normal University, Guangzhou, 510006 China; 8 Department of Counseling, Faculty of Humanities and Social Sciences, Ardakan University, Ardakan, Iran; 9 Department of Counselling, Faculty of Humanities, University of Allameh Tabataba’i, Tehran, Iran; 10 Department of Neuroscience, School of Advanced Technologies in Medicine, Mazandaran University of Medical Sciences, Sari, Iran; 11 Center for Health Related Social and Behavioral Sciences Research, Shahroud University of Medical Sciences, Shahroud, Iran

**Keywords:** Factor analysis, Food addiction, Psychometrics, Yale food addiction scale

## Abstract

**Background:**

The Modified Yale Food Addiction Scale 2.0 (mYFAS 2.0) was developed with the primary objective of evaluating food addiction (FA). The present study aimed to undertake the translation, pilot testing, and evaluation of the psychometric properties of the mYFAS 2.0 within the Persian-speaking population.

**Methods:**

The transcultural adaptation of the mYFAS 2.0 to the Persian language was conducted. Data collection was carried out through an anonymous online questionnaire. Participants completed the Persian versions of the mYFAS 2.0, Binge Eating Scale (BES), Barratt Impulsivity Scale (BIS-11), and Connor-Davidson Resilience Scale (CD-RISC). The assessment encompassed the evaluation of internal consistency reliability, factor structure, as well as convergent and discriminant validity of the aforementioned questionnaires.

**Results:**

Confirmatory factor analysis revealed that the single-factor model of the Persian translation of mYFAS 2.0 performed satisfactorily, with comparative fit index (CFI) and Tucker-Lewis index (TLI) values exceeding 0.95, standardized root mean square residual (SRMR) less than or equal to 0.09, and root mean square error of approximation (RMSEA) below 0.03. The internal consistency and composite reliability of the mYFAS 2.0 were favorable in the entire sample, as well as in both male and female groups, with alpha (α) values of 0.83, ordinal alpha (αord) of 0.93, and composite reliability (CR) of 0.86. Additionally, significant relationships were observed between the total score of BES (r = 0.59, *p* < 0.001), BIS-11 (r =  − 0.16, *p* < 0.001), and CD-RISC (r = 0.22, *p* < 0.001) with mYFAS 2.0-diagnosed FA presence, severity, and symptom count.

**Conclusions:**

The Persian version of the mYFAS 2.0 exhibited satisfactory psychometric properties.

**Supplementary Information:**

The online version contains supplementary material available at 10.1186/s40337-023-00962-1.

## Abstract (plain language summary)

In this study, researchers developed a Persian version of the Modified Yale Food Addiction Scale 2.0 (mYFAS 2.0) to assess food addiction in Persian-speaking individuals. They translated and tested the scale's reliability and validity through an online survey with 9606 Persian speaking participants. The results showed that the Persian mYFAS 2.0 performed well, with a reliable single-factor model. The internal consistency and reliability were good across the entire sample and in both male and female groups. The relationships between mYFAS 2.0 and other scales measuring binge eating, impulsivity, and resilience were significant. The findings suggest that the Persian version of mYFAS 2.0 is a reliable tool for assessing food addiction in the Persian-speaking population. The study used statistical analyses like confirmatory factor analysis, indicating the scale's robustness. Overall, the psychometric properties of the Persian mYFAS 2.0 were satisfactory, providing a valuable instrument for researchers and healthcare professionals studying and addressing food addiction in this population. The study contributes to cross-cultural research and enhances our understanding of food addiction in diverse linguistic communities.

**Supplementary Information:**

The online version contains supplementary material available at 10.1186/s40337-023-00962-1.

## Introduction

Addictive-like eating has emerged as a significant global health concern, leading to physical and psychological impairments. Previous studies have suggested that certain types of food, particularly those high in sugar and fat, may have addictive properties [1]. Food addiction (FA) refers to the addictive behavior associated with specific foods, leading to clinically significant impairment or distress. The Yale Food Addiction Scale (YFAS) was developed as the first validated instrument to assess FA, based on the Diagnostic and Statistical Manual of Mental Disorders (DSM) criteria for substance abuse [2]. Since its inception, the YFAS has been widely utilized in FA research and is considered the gold standard for studies in this field. To account for revisions in the DSM's indicators for substance dependence, a new version of the YFAS, known as YFAS version 2.0, was introduced [3]. The YFAS 2.0 incorporates additional items pertaining to craving, continued use despite negative social consequences, failure to fulfill obligations, and use in hazardous situations [4]. Recently, a shortened 13-item version of the YFAS 2.0, called the modified YFAS 2.0 (mYFAS 2.0), was developed as a more concise assessment tool for addictive-like eating. The mYFAS 2.0 includes one item for each of the eleven diagnostic indicators, along with two items to evaluate clinically significant impairment or distress. Similar to the original YFAS, the mYFAS 2.0 can be scored either as a continuous symptom count ranging from zero to eleven or as a categorical diagnosis of FA [5]. The psychometric properties of the mYFAS 2.0 closely resemble those of the YFAS 2.0, making it a valuable instrument in populations where minimizing participant burden is crucial or as a brief screening tool to identify individuals at high risk for further assessments.

The mYFAS 2.0 has been successfully translated into various languages, such as Italian [6], French [7], Czech [8], Arabic [9, 10], Brazilian Portuguese [11], Spanish [12], Turkish [13], Malay [14], traditional Chinese [15, 16], and simplified Chinese [17, 18]. Regarding Persian translations of the earlier YFAS [19] and YFAS 2.0 [20], A Persian version of the short form mPYFAS 2.0 that suits epidemiological purposes in Persian culture is lacking. Therefore, the primary objective of this research was to investigate the validity of the Persian translation of the mYFAS 2.0 in a non-clinical sample. Furthermore, the study aimed to assess the psychometric characteristics of the Persian version by conducting confirmatory factor analysis to determine its factor structure, evaluating internal consistency, and examining construct validity through associations with measures of binge eating. Additionally, the study sought to establish convergent and discriminant validity by examining the relationships between the Persian mYFAS 2.0 and measures of impulsive and resilient behaviors.

## Materials and methods

### Design and participants

In this cross-sectional study, data were collected from a sample of 9606 participants from the general population, ranging in age from 18 to 65 years. The current study was conducted online, reaching participants in all 31 provinces of Iran, as well as Persian speakers residing in other countries across all continents. Access was facilitated through social media platforms. The sample was obtained by sharing the survey link from 2021 to 2022 through various channels, including outreach via the WhatsApp messaging app, social media paid advertisements, and promotion on platforms such as Facebook®, Instagram®, Telegram, and Twitter. Prospective participants were exposed to the survey through targeted ads displaying the survey poster. All participants met the age criteria of 18–65 years and were either residents of the 31 states within Iran or Iranian immigrants residing in other countries. The study sample was selected based on the following criteria: participants were required to be over 18 years old, possess the ability to read in the Persian language, and be fluent in Persian. The information was gathered through an online questionnaire, which included inquiries about demographic characteristics such as sex, age, marital status, and education. Additional items were asked regarding smoking status. Moreover, participants completed the modified Persian version of the YFAS 2.0 (mPYFAS 2.0) as well as three validated Persian scales: the Binge Eating Scale (BES), the Barratt Impulsiveness Scale (BIS-11), and the Connor-Davidson Resilience Scale (CD-RISC). It is important to note that the study adhered to the ethical principles outlined in the Declaration of Helsinki and the Ethical Guidelines for Medical and Health research. Approval for the study was obtained from the local Ethics Committee.

Determining the appropriate sample size for conducting factor analysis is not an exact science, and there is no universally agreed-upon minimum requirement. However, it is generally recognized that larger sample sizes tend to yield more accurate and stable results. In the literature, suggested sample sizes for factor analysis vary significantly, with the recommended ratio of measured variables to subjects ranging from 1:10 to 1:2. Expert opinions and experiences suggest that a sample size of around 300 is considered good, 500 is considered very good, and 1000 or more is considered excellent for conducting factor analysis [21]. In the present study, the sample size of 9606 participants was deemed adequate based on these recommendations and guidelines. By having a large sample size, the study can provide more robust and reliable results, increasing the generalizability and statistical power of the findings.

### Measurements

#### Modified Persian YFAS 2.0 (mPYFAS 2.0)

The mPYFAS consists of 13 items that are scored on an 8-point Likert scale, ranging from zero (Never) to seven (Daily). These items assess the 11 criteria for substance use disorder outlined in the DSM-5, along with two additional items that measure the clinical significance of these symptoms (distress/impairment) (see Table 1). Each item has a different cut-off value to determine whether the criterion is met or unmet. A score of 1 is assigned if the criterion is met, and a score of 0 is assigned if it is unmet (see Table 1). Two sum scores can be calculated based on the sub-scores of the symptom criteria and the clinical significance criteria. The symptom count score ranges from 0 to 11 and represents the total number of symptoms reported. The clinical significance score ranges from 0 to 2 and indicates whether the clinical significance criterion is met. If the clinical significance score is 1 or 2, the criterion is considered met and receives a score of 1. Otherwise, if the score is 0, the criterion is unmet and receives a score of 0. To diagnose FA, the threshold is set at reporting two or more symptoms (symptom count score ranging from 2 to 11) and meeting the clinical significance criterion. Additionally, based on the number of symptoms and the presence of the clinical significance criterion, FA can be further categorized as mild (symptom count score 2–3), moderate (symptom count score 4–5), or severe (symptom count score 6–11) [5]. The internal consistency of the mPYFAS 2.0, including the 11 diagnostic indicators and the significant discomfort item, was found to be acceptable with a KR-20 coefficient of 0.77 and a McDonald omega coefficient of 0.78. These coefficients indicate the reliability and consistency of the scale in measuring FA symptoms and clinical significance.

**Table 1:** Description of the 13 items in mPYFAS 2.0 with original 8-point Likert Scale and transformed scale values (polytomous and dichotomous)

Item no.	Item descriptions	8-point likert scale (0–7)		DSM-5 criteria
		Polytomous	Dichotomous	
1	I ate to the point where I felt physically ill	0 = Never to 7 = Daily	(0–3 = 0;4–7 = 1)	Amout
2	I spent a lot of time feeling sluggish or tired from overeating	0 = Never to 7 = Daily	(0–4 = 0;5–7 = 1)	Time
3	I avoided work, school or social activities because I was afraid I would overeat there	0 = Never to 7 = Daily	(0–1 = 0;2–7 = 1)	Activities
4	If I had emotional problems because I hadn’t eaten certain foods, I would eat those foods to feel better	0 = Never to 7 = Daily	(0–3 = 0;4–7 = 1)	Withdrawal
5	My eating behavior caused me a lot of distress	0 = Never to 7 = Daily	(0–4 = 0;5–7 = 1)	Impairment
6	I had significant problems in my life because of food and eating. These may have been problems with my daily routine, work, school, friends, family, or health	0 = Never to 7 = Daily	(0–4 = 0;5–7 = 1)	Impairment
7	My overeating got in the way of me taking care of my family or doing household chores	0 = Never to 7 = Daily	(0–1 = 0;2–7 = 1)	Obligations
8	I kept eating in the same way even though my eating caused emotional problems	0 = Never to 7 = Daily	(0–3 = 0;4–7 = 1)	Consequences
9	Eating the same amount of food did not give me as much enjoyment as it used to	0 = Never to 7 = Daily	(0–4 = 0;5–7 = 1)	Tolerance
10	I had such strong urges to eat certain foods that I couldn’t think of anything else	0 = Never to 7 = Daily	(0–3 = 0;4–7 = 1)	Craving
11	I tried and failed to cut down on or stop eating certain foods	0 = Never to 7 = Daily	(0–4 = 0;5–7 = 1)	Attempts
12	I was so distracted by eating that I could have been hurt (e.g., when driving a car, crossing the street, operating machinery)	0 = Never to 7 = Daily	(0–1 = 0;2–7 = 1)	Situations
13	My friends or family were worried about how much I overate	0 = Never to 7 = Daily	(0–1 = 0;2–7 = 1)	Problems

#### Binge eating scale (BES)

The BES is a self-administered questionnaire composed of 16 items: eight items that describe behavioral manifestations (for example, eating fast or consuming large amounts of food) and eight items on associated feelings and cognitions (for example, fear of not stopping eating). Each item has a response range from 0 to 3 points (0 = no severity of the BES symptoms, 3 = serious problems on the BES symptoms) [22]. Marcus et al. (1988) created a range of scores for the BES from 0 to 46 points: a score of less than 17 points indicates minimal BE problems; a score between 18 and 26 points indicates moderate BE problems, and a score of more than 27 points indicates severe BE problems. The BES has good test–retest reliability (r = 0.87, *p *< 0.001) and moderate associations with binge eating severity as measured by food records (r = 0.20–0.40, *p *< 0.05; [23]. The Persian version of the BES showed a sensitivity of 84.6% and specificity of 80.8% in identification of binge eating disorder. The test–retest reliability and internal consistency of BES were 0.71 and 0.85 respectively. The BES effectively discriminated obese persons from the normal weight subjects [24]. In the present research, the reliability of the questionnaire was calculated using the Cronbach's alpha method, resulting in 0.79.

#### Barratt impulsiveness scale (BIS-11)

The BIS-11 is widely used to assess impulsivity traits and is considered the most commonly used self-assessment scale for measuring the impulsive dimension. Over the past five decades, the BIS-11 has undergone several modifications, with the latest version developed by Patton et al. in 1995 [25]. It is rated on a four-point Likert scale of 1 = Rarely/Never to 4 = Almost Always/Always. The total scores can range from 30 to 120 [26]. 12 items are reverse scored to account for response biases. The scale includes three second-order factors: attentional, motor, and non-planning impulsiveness. Higher scores on the BIS-11 indicate lower levels of attention, increased hyperactivity, and a lack of planning. The effectiveness of the BIS-11 in evaluating impulsive dimensions has been well-established [27]. This questionnaire was validated in the Aryan population by Javid et al. in 2012. Five questions, specifically numbered 29, 23, 20, 17, and 6, were excluded. The remaining 25 questions demonstrated appropriate validity and reliability. Among these questions, items 29 and 20 are reverse-scored [28]. In the present study, the 25-item form of the Persian BIS-11 was utilized. The internal consistency of the Persian BIS-11 has been reported to be highly acceptable, with a Cronbach's alpha coefficient of 0.81. In the present study, the reliability of the questionnaire was calculated using the Cronbach's alpha method, resulting in 0.86. This indicates the scale's reliability and consistency in measuring impulsivity traits among individuals in the Persian-speaking population. To calculate the score for each subscale, sum the scores of its respective questions. To obtain the overall questionnaire score, sum the scores of all questions together. A higher score in this questionnaire indicates low impulsivity and high behavioral inhibition, while a lower score suggests experiencing high impulsivity. The highest achievable score in this questionnaire is 100, and the lowest is 25.

#### Connor-Davidson resilience scale (CD-RISC)

The CD-RISC has 25 items and five subscales of personal competence, tolerance of negative affect, positive acceptance, self-control, and spiritual influences responded based on a 5-point Likert scale (completely false = 0 to completely true = 4). The score range on this scale is 0–100, and a high score is suggestive of more resilience [29]. This scale was run and validated in the general and clinical population (people with Generalized Anxiety Disorder and PTSD) by Connor et al., 2003, demonstrating good psychometric properties and obtaining the factor analysis of five factors. Moreover, repeated-measures ANOVA revealed that the patient’s further improvement during treatment was associated with an increase in CD-RISC score; furthermore, in the clinical sample, the test–retest reliability of this scale was calculated to be 0.87 [29]. In Iran, this scale was standardized by Bakhsayesh Eqbali and colleagues in 2022 [30]. The confirmatory factor analysis results of the first stage with five factors revealed that 25 CD-RISC items benefited from high factor load and good fit indices were reported (χ^2^ = 605.55; df = 265; *P* Value = 0.0001; χ^2^/df = 2.28; GFI = 0.88; CFI = 0.93; TLI = 0.92; RMR = 0.06; RMSEA = 0.05); therefore, CD-RISC has good construct validity. Cronbach’s alpha coefficients for the whole scale and subscales were calculated at 0.94 and 0.71–0.89, respectively, indicating the optimal reliability of CD-RISC in PwMS. In the current study, the reliability of the questionnaire was calculated using the Cronbach's alpha method, resulting in 0.83.

### Statistical analyses

The collected data were subjected to various statistical analyses to investigate the validity and reliability of the mYFAS 2.0 diagnostic indicators. Descriptive statistics, such as frequency percentages, minimum and maximum scores, averages, and standard deviations, were calculated for each item. The internal consistency of the diagnostic indicators was assessed using Kuder-Richardson alpha (KR-20) and McDonald's omega coefficients.

To evaluate the structural validity of the mYFAS 2.0, confirmatory factor analysis (CFA) was employed. This analysis focused solely on the diagnostic indicators and did not consider clinically important impairment or discomfort. The fit of the model was assessed using indices such as χ^2^ divided by degrees of freedom (χ^2^/df), Comparative Fit Index (CFI), Tucker–Lewis Index (TLI), Root Mean Squared Error of Approximation (RMSEA), and Standardized Root Mean Square Residual (SRMR). The model fits the data well when: χ^2^/df ≤ 3, CFI and TLI ≥ 0.95, RMSEA < 0.06 and SRMR < 0.08 [31, 32]. An acceptable model fit is indicated by χ^2^/df ≤ 5, CFI and TLI ≥ 0.90, RMSEA < 0.08 and SRMR < 0.1 [33].

These indices provide information on how well the observed data align with the hypothesized factor model. For the statistical analyses, R software (version 4.2.2) with the psych package was used for descriptive analysis. MPLUS software (version 8.3) was employed for confirmatory factor analysis, utilizing the WLSMV (weighted least squares mean and variance adjusted) estimation method suitable for rank data. Microsoft Excel was used to calculate the reliability coefficient of Kuder-Richardson alpha (KR-20) [34], and JASP software (version 34) was used to calculate McDonald's omega [35, 36]. The significance level was set at *p *< 0.05 to determine statistical significance. By conducting these analyses, the study aimed to provide comprehensive insights into the validity and reliability of the mYFAS 2.0 diagnostic indicators in assessing FA.

## Results

### Sample characteristics

The demographic characteristics of the 9606 participants are presented in Table 2.

**Table 2:** Subject characteristics (n = 9606)

Characteristics	Frequency n (%)
Age (year) ^*^	29.61 (9.00)
*Sex*	
Female	7749 (80.7)
Male	1857 (19.3)
*Years of Education*	
1–10	162 (1.7)
11–15	3433 (35.7)
16–20	5576 (58)
21–24	435 (4.5)
*Marriage status*	
Married	5214 (54.3)
Single	4053 (42.2)
Other	339 (3.5)

*Mean (± SD)

The descriptive information of each item in polytomous scores version (range 0–7), such as the ratio of responses to each option, mean, standard deviation, and ratio of correct answers in polytomous scores version, is presented in Table 3. Items 5 and 6 were not included since they are two impairment and distress questions, which are different from the remaining 11 symptom items. Please note that the remaining tables present the statistics based on these FA 11 items.

**Table 3:** Descriptive indicators of food addiction scale in 11 item version

Item	Response ratio to options								Mean	SD
	0	1	2	3	4	5	6	7		
1	0.36	0.19	0.10	0.13	0.07	0.07	0.04	0.03	1.89	2.03
2	0.35	0.20	0.09	0.13	0.06	0.08	0.04	0.03	1.93	2.06
3	0.92	0.04	0.01	0.01	0.01	0.01	0.00	0.01	0.22	0.94
4	0.50	0.15	0.08	0.11	0.06	0.05	0.03	0.02	1.46	1.93
7	0.86	0.05	0.02	0.02	0.01	0.02	0.01	0.01	0.40	1.26
8	0.64	0.11	0.04	0.06	0.02	0.04	0.03	0.05	1.22	2.08
9	0.56	0.13	0.06	0.08	0.03	0.05	0.03	0.06	1.44	2.14
10	0.38	0.20	0.11	0.13	0.05	0.06	0.03	0.03	1.76	1.98
11	0.46	0.16	0.10	0.12	0.04	0.05	0.03	0.05	1.64	2.06
12	0.93	0.03	0.01	0.01	0.00	0.00	0.00	0.00	0.15	0.73
13	0.67	0.11	0.06	0.06	0.02	0.03	0.01	0.04	1.00	1.87

Table 4 presents the correlations between each item and the total score when the specific item is removed in both the polytomous and dichotomous modes. The correlations reported in the table are all above 0.30, indicating a relatively strong relationship between each item and the total score [37].

**Table 4:** Correlation of items with the total score in polytomous and dichotomous version of FA 11 items

Items	Polytomous version	Dichotomous version
1	0.67	0.72
2	0.73	0.82
3	0.53	0.52
4	0.65	0.73
7	0.76	0.76
8	0.82	0.89
9	0.54	0.64
10	0.69	0.81
11	0.70	0.79
12	0.57	0.61
13	0.71	0.72

Additionally, the correlations between the score of each item and the total score of other items, excluding the item itself, in both the polytomous and dichotomous versions demonstrate a positive and significant relationship. This suggests that each item is closely related to the overall score and reflects the collective nature of the items in assessing the construct of interest. These findings provide evidence for the good relationship and similarity between the individual items and the total score, indicating the coherence and consistency of the items in measuring the construct being assessed.

Descriptive indicators of the total scale score in the whole sample and by gender in polytomous and dichotomous version are presented in Table 5. The independent t-test indicates that there is a significant difference between women and men in polytomous and dichotomous version, and this difference is in favor of women.

**Table 5:** Mean, standard deviation, minimum, and maximum of the test for the whole sample and by gender in polytomous and dichotomous version of FA 11 items

Data type	Sample	Skewness	Kurtosis	Mean	Standard deviation	*T*
Polytomous	Total	1.5	2.13	13.11	13.09	–
	Female	1.44	1.86	13.72	13.4	9.494**
	Male	1.73	3.57	10.53	11.36	
Dichotomous	Total	1.82	2.75	1.5	2.29	–
	Female	1.74	2.35	1.57	2.36	6.178**
	Male	2.18	5.05	1.21	1.94	

**p < .01

Table 6 presents the fit indices for the single-factor structure of the scale for the total sample, as well as for women and men separately, in both the polytomous and dichotomous versions of the data. The results indicate that the single-factor model demonstrates a good fit with the data in the total sample for both the polytomous and dichotomous versions. However, it is worth noting that the fit of the model is slightly better in the dichotomous version compared to the polytomous version, as indicated by the RMSEA index. The fit indices suggest that the single-factor model adequately captures the underlying structure of the scale in both data versions for the total sample. Furthermore, when examining the fit of the model separately for women and men, similar findings are observed. The model demonstrates a good fit with the data for both polytomous and dichotomous versions in both gender groups. Once again, the fit of the model is slightly better in the dichotomous version compared to the polytomous version. These results indicate that the single-factor structure of the scale is well-supported by the data, suggesting that the items of the scale are measuring a common underlying construct. The findings also suggest that the dichotomous version of the data provides a better fit to the single-factor model compared to the polytomous version.

**Table 6:** Fit indices of the one-factor model by gender and total sample of FA 11 items

Data type	Sample	χ^2^	df	TLI	CFI	RMSEA [CI 90%]	SRMR
Polytomous	Total	3486.848	44	0.948	0.959	0.090 [0.088–0.093]	0.034
	Female	2776.023	44	0.953	0.962	0.090 [0.087–0.092]	0.031
	Male	653.664	44	0.929	0.943	0.086 [0.081–0.092]	0.041
Dichotomous	Total	1005.346	44	0.975	0.980	0.048 [0.045–0.050]	0.047
	Female	780.127	44	0.978	0.983	0.046 [0.044–0.049]	0.044
	Male	224.469	44	0.958	0.955	0.047 [0.041–0.053]	0.063

Ordinal alpha, combined reliability, and discriminant validity statistics for the entire sample, men and women, in Table 7 show that the level of internal consistency and composite reliability in the entire sample and male and female groups is favorable. Based on the AVE index, whose values greater than 0.5 are usually acceptable, convergent validity is also acceptable; its value is higher in dichotomous data than in polytomous version. Therefore, in the polytomous version, it can be observed that the structural model explains approximately 51% of the variance in the total sample, with slightly lower values of 49% for men and 52% for women. In the dichotomous version, the structural model accounts for approximately 59% of the variance in the total sample, with values of 56% for men and 59% for women. Overall, the explained variance is generally higher in women compared to men. To establish the construct-level discriminant validity, it is necessary to examine whether the square root of the Average Variance Extracted (AVE) index exceeds the correlation between the FA variable and other variables (Table 6) [38].

**Table 7:** Alpha statistics, combined reliability and diagnostic validity of AVE by the whole sample, women and men of FA 11 items

Data type	Group	α	α_ord_	Composite reliability^a^	AVE
Polytomous	Total	0.871	0.915	0.895	0.510
	Male	0.846	0.908	0.877	0.490
	Female	0.875	0.917	0.898	0.516
Dichotomous	Total	0.836	0.935	0.865	0.586
	Male	0.797	0.936	0.832	0.555
	Female	0.842	0.937	0.870	0.593

^a^Composite reliability

BES, BIS, and CD-RISC scales were significantly associated with the mYFAS 2.0 diagnosis (Table 8). There was a significant correlation between the BES, BIS, and CD-RISC scales and the mYFAS 2.0-diagnosed FA symptom count.

**Table 8:** Spearman’s rank correlation coefficients among the mYFAS 2.0-diagnosed food addiction (FA) symptom count (13 items), BES (25 items), CD-RISC (25 ietms), and BIS-11 (25 items) total score

	FA Symptom count	BES	CD-RISC	BIS-11
FA symptom count	1			
Binge eating scale (BES)	0.594^**^	1		
Connor-Davidson resilience scale (CD-RISC)	− 0.166^**^	− 0.254^**^	1	
Barratt impulsiveness scale-11 (BIS-11)	0.226^**^	0.338^**^	− 0.481^**^	1

**< .01

### Examining invariance in the 11-items test

To examine the factor structure invariance based on gender, four models were assessed: Configural invariance, metric or weak invariance, scalar or strong invariance, and exact invariance. The analysis aimed to determine if the factor structure of FA was consistent across male and female groups. In the Configural invariance model, no restrictions were imposed on any parameters, and the same factorial structure was fitted to both groups. A significant fit indicated that the factor structure was equivalent in both groups. The metric model assumed equality of factor loadings between the groups. Lack of significance in the comparison between the metric and Configural models suggested invariance, indicating that the construct had the same meaning for both groups. This implied that the items captured the same underlying construct, allowing for comparison of variance and covariance of scores between the groups. The scalar model, considering the ordinal nature of the data, enforced equality of item thresholds in addition to factor loadings. Lack of significance in the comparison between the scalar and metric models allowed for comparing the means of the latent variable between the groups. In the exact model, residual variances of the items were assumed to be equal between the groups. Lack of significance in the comparison between the exact and scalar models allowed for comparing the total scores between the groups based on the sum of observed item scores. This indicated that the reliability of the items was consistent between the two groups. Finally, the variance and mean of the latent structure among the groups were examined. The analysis was conducted using Mplus software, employing the WLSMV estimation method suitable for ranked data (response range of 0 to 7 in the polytomous version). Thus, the models were fitted based on the polychoric correlation matrix. Model comparison was performed using the DIFTEST method in Mplus, considering chi-square difference test, as well as fit indices such as CFI, TLI, SRMR, and RMSEA to evaluate the differences between the models (Table 9).

**Table 9:** Fit indices of different models to check invariance in polytomous items

Model	${\mathrm{\rm X}}^{2}$	df	*P*	CFI	TLI	SRMR	RMSEA [CI 90%]	$\Delta {\mathrm{\rm X}}^{2}$	dfΔ	*P*	CFIΔ	TLIΔ	RMSEAΔ	RMRΔ
Configural model	3725.869	100	0.0001	0.956	0.951	0.038	[0.085–0.089] 0.087	–	–	–	–	–	–	–
Metric 1	2844.028	110	0.0001	0.967	0.967	0.039	[0.070–0.074] 0.072	44.260	10	0.0001	− 0.011	− 0.016	0.015	− 0.001
Metric 2	2950.716	109	0.0001	0.965	0.965	0.039	[0.071–0.076] 0.074	26.395	9	0.0018	0.002	0.002	− 0.002	0.000
Metric 3	3147.753	108	0.0001	0.963	0.962	0.038	[0.074–0.079] 0.077	14.683	8	0.0656	0.002	0.003	− 0.003	0.001
Scalar 1	1555.588	172	0.0001	0.969	0.980	0.037	[0.055–0.061] 0.058	160.219	64	0.0001	− 0.006	− 0.018	0.019	0.001
Scalar 2	1502.196	171	0.0001	0.970	0.981	0.036	[0.054–0.060] 0.057	109.408	63	0.0003	− 0.001	− 0.001	0.001	0.001
Scalar 3	1456.417	170	0.0001	0.971	0.981	0.037	[0.053–0.059] 0.056	63.370	62	0.4278	− 0.001	0.000	0.001	− 0.001
Strict 1	1444.787	159	0.0001	0.971	0.980	0.035	[0.055–0.061] 0.058	–	–	–	0.000	0.001	− 0.002	0.002
Strict 2	1456.417	170	0.0001	0.971	0.981	0.037	[0.053–0.059] 0.056	110.341	11	0.0001	0.000	− 0.001	0.002	− 0.002
Strict 3	1390.430	169	0.0001	0.973	0.982	0.036	[0.052–0.058] 0.055	71.817	10	0.0001	− 0.002	− 0.001	0.001	0.001
Strict 4	1353.148	168	0.0001	0.974	0.983	0.036	[0.052–0.057] 0.054	47.990	9	0.0001	− 0.001	− 0.001	0.001	0.000
Strict 5	1341.050	167	0.0001	0.974	0.983	0.036	[0.051–0.057] 0,054	35.850	8	0.0001	0.000	0.000	0.000	0.000
Strict 6	1342.876	166	0.0001	0.974	0.983	0.036	[0.052–0.057] 0,054	27.477	7	0.0003	0.000	0.000	0.000	0.000
Strict 7	1363.498	165	0.0001	0.973	0.982	0.035	[0.052–0.058] 0.055	21.556	6	0.0015	0.001	0.001	− 0.001	0.001
Strict 8	1350.710	164	0.0001	0.973	0.982	0.035	[0.052–0.058] 0.055	11.180	5	0.0479	0.000	0.000	0.000	0.000
Strict 9	1348.300	163	0.0001	0.974	0.982	0.035	[0.052–0.058] 0.055	5.438	4	0.2452	− 0.001	0.000	0.000	0.000
Variance	1066.472	164	0.0001	0.980	0.986	0.035	[0.045–0.051] 0.048	4.150	1	0.0416	− 0.006	− 0.004	0.007	0.000
Mean	1557.366	164	0.0001	0.969	0.979	0.037	[0.057–0.062] 0.059	59.600	1	0.0001	0.011	0.007	− 0.011	− 0.002

### Polytomous data

According to the findings presented in Table 9, a comparison between the Configural model and metric model 1 revealed a significant difference (*p *< 0.05). Further examination of modification indices indicated that the factor loadings of items 12 and 3 differed significantly between the two groups. Consequently, in metric models 2 and 3, these factor loadings were freely estimated for the respective groups. As a result, the difference between metric model 3 and the Configural model became non-significant (*p *> 0.05). Similarly, when comparing scalar model 1 with metric model 3, it was observed that the first threshold of item 4 and the second threshold of item 13 differed between men and women (*p *< 0.05). In scalar models 2 and 3, these thresholds were freely estimated for the respective groups. Consequently, the difference between scalar model 3 and metric model 3 became non-significant (*p *> 0.05).

In strict model 1, the variance of the remaining items was freely estimated between the two groups, while in strict model 2, this parameter was constrained to be the same across the two groups. The results indicated a statistically significant chi-square difference between the two models (*p *< 0.05). Subsequently, in strict models 2–9, the error variances of items 13, 1, 4, 7, 12, 2, and 11 were freely estimated between the two groups. The comparison of strict model 9 with strict model 1 yielded a non-significant chi-square difference (*p *> 0.05). Moreover, the last two rows of Table 8 demonstrate that while the mean and variance of the women's group (reference group) were fixed at zero and one, respectively, constraining these parameters in the men's group resulted in a significant chi-square difference (*p *< 0.05). This indicates that the mean and variance differ between women and men in the structure of FA. Specifically, the average score for men (target group) in the FA structure was -0.328, with a variance of 0.96. In comparison, the women's group (reference group) had scores ranging from zero to one, indicating higher mean and variance. Thus, it can be concluded that the mean and variance of the men's group are lower than those of the women's group.

The reported parameters in Table 10 are based on the results of strict model 2. Considering the sensitivity of the chi-square test to sample size, based on the ΔCFI index ≤ 0.01, it can be inferred that the most influential models in terms of gender are metric 1, scalar 1, and average models. However, based on the ΔRMSEA ≥ 0.015 and ΔSRMR ≥ 0.03 criteria, none of the identified effects are considered significant [36].

**Table 10:** Factor load, threshold values, and the remaining variance of items based on the exact model 2

Gender	item	Loading (S.E)	Threshold 1 (S.E)	Threshold 2 (S.E)	Threshold 3 (S.E)	Threshold 4 (S.E)	Threshold 5 (S.E)	Threshold 6 (S.E)	Threshold 7 (S.E)	Residual variances
Female	FA1	1.242 (0.031)	− 0.617 (0.031)	0.164 (0.031)	0.564 (0.032)	1.205 (0.036)	1.659 (0.041)	2.311 (0.048)	2.883 (0.058)	–
	FA2	1.388 (0.033)	− 0.701 (0.034)	0.192 (0.033)	0.598 (0.035)	1.254 (0.039)	1.654 (0.043)	2.433 (0.052)	3.166 (0.063)	–
	FA3	0.668 (0.041)	1.679 (0.042)	2.022 (0.048)	2.181 (0.051)	2.377 (0.056)	2.546 (0.062)	2.760 (0.070)	2.969 (0.081)	–
	FA4	0.942 (0.025)	− 0.132 (0.028)	0.454 (0.027)	0.783 (0.029)	1.280 (0.033)	1.667 (0.037)	2.141 (0.043)	2.602 (0.054)	–
	FA7	1.355 (0.056)	1.818 (0.062)	2.253 (0.069)	2.452 (0.073)	2.729 (0.078)	2.960 (0.083)	3.445 (0.095)	3.834 (0.109)	–
	FA8	1.757 (0.054)	0.675 (0.044)	1.291 (0.050)	1.578 (0.053)	1.999 (0.057)	2.238 (0.059)	2.789 (0.064)	3.204 (0.067)	–
	FA9	0.681 (0.022)	0.140 (0.023)	0.552 (0.024)	0.774 (0.025)	1.109 (0.027)	1.296 (0.028)	1.610 (0.032)	1.844 (0.035)	–
	FA10	1.099 (0.026)	− 0.523 (0.028)	0.255 (0.029)	0.692 (0.030)	1.304 (0.033)	1.616 (0.035)	2.183 (0.042)	2.674 (0.051)	–
	FA11	1.133 (0.029)	− 0.198 (0.028)	0.386 (0.030)	0.786 (0.031)	1.365 (0.036)	1.631 (0.038)	2.094 (0.043)	2.422 (0.048)	–
	FA12	0.760 (0.048)	1.883 (0.054)	2.321 (0.064)	2.512 (0.069)	2.694 (0.074)	2.892 (0.082)	3.182 (0.097)	3.457 (0.119)	–
	FA13	1.238 (0.039)	0.668 (0.035)	1.290 (0.042)	1.550 (0.043)	1.970 (0.048)	2.174 (0.051)	2.514 (0.056)	2.748 (0.060)	–
Male	FA1	1.242 (0.031)	− 0.617 (0.031)	0.031 (5.275)	0.564 (0.032)	1.205 (0.036)	1.659 (0.041)	2.311 (0.048)	2.883 (0.058)	1.554
	FA2	1.388 (0.033)	− 0.701 (0.034)	0.192 (0.033)	0.598 (0.035)	1.254 (0.039)	1.654 (0.043)	2.433 (0.052)	3.166 (0.063)	1.370
	FA3	0.843 (0.084)	1.679 (0.042)	2.022 (0.048)	2.181 (0.051)	2.377 (0.056)	2.546 (0.062)	2.760 (0.070)	2.969 (0.081)	1.000
	FA4	0.942 (0.025)	0.244 (0.048)	0.454 (0.027)	0.783 (0.029)	1.280 (0.033)	1.667 (0.037)	2.141 (0.043)	2.602 (0.054)	0.716
	FA7	1.355 (0.056)	1.818 (0.062)	2.253 (0.069)	2.452 (0.073)	2.729 (0.078)	2.960 (0.083)	3.445 (0.095)	3.834 (0.109)	1.411
	FA8	1.757 (0.054)	0.675 (0.044)	1.291 (0.050)	1.578 (0.053)	1.999 (0.057)	2.238 (0.059)	2.789 (0.064)	3.204 (0.067)	1.000
	FA9	0.681 (0.022)	0.140 (0.023)	0.552 (0.024)	0.774 (0.025)	1.109 (0.027)	1.296 (0.028)	1.610 (0.032)	1.844 (0.035)	1.000
	FA10	1.099 (0.026)	− 0.523 (0.028)	0.255 (0.029)	0.692 (0.030)	1.304 (0.033)	1.616 (0.035)	2.183 (0.042)	2.674 (0.051)	1.000
	FA11	1.133 (0.029)	− 0.198 (0.028)	0.386 (0.030)	0.786 (0.031)	1.365 (0.036)	1.631 (0.038)	2.094 (0.043)	2.422 (0.048)	0.783
	FA12	0.938 (0.092)	1.883 (0.054)	2.321 (0.064)	2.512 (0.069)	2.694 (0.074)	2.892 (0.082)	3.182 (0.097)	3.457 (0.119)	1.313
	FA13	1.238 (0.039)	0.668 (0.035)	0.830 (0.062)	1.550 (0.043)	1.970 (0.048)	2.174 (0.051)	2.514 (0.056)	2.748 (0.060)	1.764

Table 9 shows the results of the strict model 9. In this model, the mean and variance of women are fixed at zero and one, respectively, while the men's group is freely estimated with previously mentioned values. The factor loading of items 3 and 12 in the male group is freely estimated, while the factor loading of other items remains the same in both groups. The first threshold of item 4 and the second threshold of item 13 have been freely estimated for both women and men. Additionally, the error variances of items 13, 1, 4, 7, 12, 2, and 11 between the two groups were freely estimated. The factor loadings of the items, along with the seven threshold values (corresponding to the spectrum of eight items, resulting in seven threshold values for each item), as well as the variance of the remaining items in raw form, are presented in Table 8. It should be noted that in the women's group, the error variances were fixed at one, hence their specific values are not reported.

### Dichotomous data

The results presented in Table 9 indicate that the comparison between the Configural model and metric model 1 yielded a statistically significant difference (*p *< 0.05). Further analysis of the modification indices revealed that the factor loadings of items 12 and 3 significantly differed between the two groups, with item 12 having a higher modification index than item 3. Consequently, the factor loading of item 12 was freely estimated between the two groups (metric model 2). This adjustment resulted in a non-significant difference in the chi-square test between metric model 2 and the Configural model (*p *> 0.05), indicating metric invariance. Next, the invariance of threshold values for the items was examined. Comparing scalar model 1 with metric model 2 revealed significant modification indices for the threshold values of items 13, 4, and 9 between women and men (*p *< 0.05). Subsequently, the threshold parameter for item 13 (scalar model 2) and item 4 (scalar model 3) were freely estimated for both groups. The comparison between scalar model 2 and metric model 2, as well as scalar model 3 and metric model 2, did not result in a significant difference (*p *> 0.05). It is worth noting that none of the threshold values for the remaining items exhibited significant differences between the two groups, and thus, were not considered for free estimation (likely due to the large sample size). Moving forward, the remaining item values were examined for invariance.

In the strict model 1, the variance of the residual errors for the items was freely estimated between the two groups, while in the strict model 2, this parameter was constrained to be the same across the groups. The chi-square difference test between the two models did not yield a statistically significant result (*p *≥ 0.05). Further examination of the strict model 2 indicated that the highest modification indices were associated with the error covariance between items 1 and 2 (169.180) and items 4 and 10 (101.827) in women. However, these results were not observed in men. Despite these findings, the lack of significance in the chi-square test limited the free estimation of these error covariances between the two groups. Next, the invariance of the variance and mean of the latent structure was assessed between the two groups. The results from the variance and mean model (Table 7) showed that while the mean and variance of the women's group were fixed at zero and one, respectively, adjusting the variance of the men's group (the target group) with the women's group (the reference group) resulted in statistical significance (*p *< 0.05), indicating a difference in variance between the groups. The variance of the male group in the FA structure was estimated to be 1.314, which is higher than the variance of the female group. Based on the mean model results, it can be observed that setting the mean of the male group in the FA structure equal to the mean of the female group led to chi-square significance (*p *< 0.05), indicating that the mean of the male group (− 0.575) is lower than that of the female group in the FA structure.

The estimates presented in Table 11 are derived from detailed model 2. Considering the sensitivity of the chi-square test to sample size, based on a ΔCFI index ≤ 0.01, it can be concluded that gender has a limited influence in the two-value mode [39]. However, based on ΔRMSEA ≥ 0.015 and ΔSRMR ≥ 0.03, the effects related to the invariance of factor variances (that is variability in a latent variable and the relationships among multiple latent variables is equivalent across groups) and mean between the two groups are statistically significant. Table 12 provides the factor loadings of the questionnaire items in the round value mode (with a threshold for each item) and the variance estimates of the remaining items in raw mode. In the women's group, the error variances have been fixed at one, and thus their specific values are not reported.

**Table 11:** Fit indices of different models to check the invariance of the 11-items scale in dichotomous version

Model	${\mathrm{\rm X}}^{2}$	df	*P*	CFI	TLI	SRMR	RMSEA[CI90%]	$\Delta {\mathrm{\rm X}}^{2}$	dfΔ	*P*	CFIΔ	TLIΔ	RMSEAΔ	RMRΔ
Configural model	949.120	89	0.0001	0.978	0.978	0.048	[0.042–0.047] 0/045	–	–	–	–	–	–	–
Metric 1	707.830	99	0.0001	0.987	0.986	0.051	[0.033–0.038] 0.036	21.845	10	0.0159	− 0.005	− 0.008	0.009	− 0.003
Metric 2	722.435	98	0.0001	0.987	0.985	0.050	[0.034–0.039] 0.036	13.522	9	0.1404	0.000	0.001	0.000	0.001
Scalar 1	854.652	107	0.0001	0.984	0.984	0.051	[0.036–0.041] 0.038	132.759	9	0.0001	0.003	0.001	− 0.002	− 0.001
Scalar 2	778.868	106	0.0001	0.986	0.985	0.050	[0.034–0.039] 0.036	57.728	8	0.0001	− 0.002	− 0.001	0.002	0.001
Scalar 3	760.836	105	0.0001	0.986	0.986	0.050	[0.034–0.038] 0.036	39.934	7	0.0001	0.000	− 0.001	0.000	0.000
Strict 1	923.859	94	0.0001	0.983	0.980	0.049	[0.040–0.045] 0.043	–	–	–	0.003	0.006	− 0.007	0.001
Strict 2	760.836	105	0.0001	0.986	0.986	0.050	[0.034–0.038] 0.036	18.627	11	0.0001	− 0.003	− 0.006	0.007	− 0.001
Variance	643.854	106	0.0001	0.980	0.980	0.035	[0.045–0.051] 0.048	10.170	1	0.0014	0.006	0.000	− 0.012	0.015
Mean	756.140	106	0.0001	0.986	0.986	0.051	[0.033–0.038] 0.036	12.551	1	0.0004	− 0.006	0.000	0.012	− 0.016

**Table 12:** Factor loadings, threshold values, and the remaining variance of items based on exact model 1

Gender	Item	Loading (S.E)	Threshold1(S.E)	Residual variances
Female	FA1	1.29 (0.043)	1.249 (0.036)	–
	FA2	1.822 (0.068)	1.990 (0.065)	–
	FA3	0.605 (0.037)	1.946 (0.041)	–
	FA4	1.250 (0.043)	1.497 (0.039)	**–**
	FA7	1.230 (0.053)	2.137 (0.061)	**–**
	FA8	2.402 (0.108)	2.600 (0.107)	**–**
	FA9	0.891 (0.034)	1.441 (0.033)	**–**
	FA10	1.667 (0.058)	1.697 (0.052)	**–**
	FA11	1.475 (0.054)	1.938 (0.056)	**–**
	FA12	0.786 (0.053)	2.417 (0.067)	**–**
	FA13	1.122 (0.037)	1.204 (0.032)	**–**
Male	FA1	1.299 (0.043)	1.249 (0.036)	2.255
	FA2	1.822 (0.068)	1.990 (0.065)	2.388
	FA3	0.605 (0.037)	1.946 (0.041)	1.205
	FA4	1.250 (0.043)	1.798 (0.147)	1.622
	FA7	1.230 (0.053)	2.137 (0.061)	1.621
	FA8	2.402 (0.108)	2.600 (0.107)	1.943
	FA9	0.891 (0.034)	1.441 (0.033)	1.412
	FA10	1.667 (0.058)	1.697 (0.052)	1.611
	FA11	1.475 (0.054)	1.938 (0.056)	1.467
	FA12	1.085 (0.104)	2.417 (0.067)	1.671
	FA13	1.122 (0.037)	0.577 (0.122)	1.638

## Discussion

This study aimed to translate, pilot, and evaluate the psychometric properties of the translated version of the YFAS 2.0 in a non-clinical sample of Persian-speaking individuals. Our findings demonstrated satisfactory internal consistency reliability, factor structure, as well as convergent and discriminant validity of the translated tool. The present research also confirmed robust psychometric properties of the Persian version of the modified YFAS 2.0 (mYFAS 2.0), which were consistent with the findings from previous studies conducted in English, Czech, French, Italian, and Brazilian Portuguese-speaking populations [5–8]. The results indicated that the Comparative Fit Index (CFI) was 0.95, the Root Mean Square Error of Approximation (RMSEA) was 0.03, and the factor loadings for all diagnostic criteria, along with the significant discomfort factor of the mPYFAS 2.0, were deemed acceptable. Comparable investigations by Brunault et al. (CFI = 1.00, RMSEA = 0.0090%)^6^ and Imperatori et al. (CFI = 0.905, RMSEA = 0.086) have reported the psychometric properties of the French and Italian versions of the modified YFAS 2.0, respectively, in non-clinical populations. In both studies, all factor loadings exceeded 0.5, suggesting good model fit and reliability [7].

Item 2 exhibited the highest average score, indicating that it was rated more favorably by participants on average, while item 12 had the lowest average score, indicating less favorable ratings. Furthermore, item 9 displayed the highest dispersion, indicating greater variation in participants' responses, whereas item 12 exhibited the smallest dispersion, suggesting less variability in responses. Specifically, for item 12, a smaller proportion of participants selected items 1–7. Notably, items 3, 7, and 12 had a substantial number of participants selecting zero items. Consequently, the reduced number of individuals exhibiting specific characteristics related to these items is observed in the dichotomous version of these items.

As widely recognized, in both the polytomous and dichotomous versions, women exhibit higher mean scores and standard deviations in the context of food addiction compared to men. This difference is statistically significant, with a p-value less than 0.05. While the model demonstrates an acceptable fit with the data for the entire sample and each gender subgroup in the case of polytomous data, the model fit is generally superior for the dichotomous data. Specifically, within the women subgroup, the model fit is notably better than that observed within the men subgroup.

The composite reliability for both the polytomous and discriminant versions exceeds 0.70, indicating satisfactory measurement accuracy of the tool. Moreover, the structure of FA accounts for a minimum of 50% of the indicator variance, as indicated by the Average Variance Extracted (AVE) index. This level of explanation is deemed acceptable, given the higher composite reliability value of 0.7. Additionally, by comparing the square root of AVE with the correlation of the FA structure with other variables presented in Table 6, it can be inferred that the FA structure exhibits diagnostic validity.

The examination of a data-filled model with separate groups indicated that the factor structure of the FA construct remains consistent between men and women. However, comparing the metric and shape models revealed that the meaning of the structure differs between the male and female groups. Specifically, caution should be exercised when comparing the two groups based on the observed score variance, as items 3 and 12 exhibit distinct meanings between men and women. Notably, the divergence in the first threshold of item 4 and the second threshold of item 13 between the male and female groups indicates a difference in the latent variable's average between the two groups, with items 4 and 13 significantly contributing to this disparity. Moreover, the discrepancy in error variances for items 13, 1, 4, 7, 12, 2, and 11 between the male and female groups suggests that the reliability of these items varies between the two groups. Therefore, it is preferable to evaluate the two groups based on the observed score. Comparing the mean and variance of the FA structure between men and women reveals that men have lower mean and variance values compared to women. Based on the ΔCFI index (≤ 0.01), gender exerts the greatest influence on metric 1, scalar 1, and the average model. However, according to the ΔRMSEA (≥ 0.015) and ΔSRMR (≥ 0.03) indices, none of the identified effects are considered significant.

The shape model analysis of bivariate data suggests that the factor structure of FA is similar between the two groups. In the metric model applied to dichotomous data, the only difference observed between the two groups was in the factor loading of item 12. Similarly, in the scalar model, the threshold values of items 13 and 4 were found to be different between the two groups. However, based on the exact model, the residual variances were found to be equivalent across the two groups. Comparing the variance and mean of the FA structure between men and women, it is evident that men have higher variance but lower mean in this structure compared to women. It is noteworthy that the influence of gender in the two-value mode, as indicated by the ΔCFI index (≤ 0.01), is also low according to Chang and Rensold (2002). However, the effects related to the invariance of factor variances (that is variability in a latent variable and the relationships among multiple latent variables is equivalent across groups) and mean between the two groups are found to be significant based on the ΔRMSEA (≥ 0.015) and ΔSRMR (≥ 0.03) indices.

The convergent validity indicators in this study align with previous literature on the YFAS 2.0 and mYFAS 2.0, as evidenced by their correlation with binge eating [7, 40–43]. Our findings revealed a strong positive correlation between mPYFAS 2.0 and BES scores. Similarly, Brunault et al. observed a correlation between FA and BES scores in their validation study of the French version of mYFAS 2.0^(7)^. Imperatori et al. also reported a similar result regarding the convergent validity of the Italian version of YFAS 2.0, demonstrating a correlation with BES scores [5]. Moreover, consistent with the Brazilian mYFAS 2.0, our study demonstrated an association between mPYFAS 2.0 and impulsivity, as measured by the BIS. Extensive evidence highlights the correlation between FA and impulsivity [44]. Additionally, Pivarunas et al. found that negative urgency, impulsiveness, and emotion dysregulation could predict the symptom count on the YFAS [45]. Studies exploring the relationship between overeating and the BIS have reported that specific dimensions of impulsivity are correlated with certain FA symptoms [38].

To evaluate the discriminant validity of the Persian mYFAS 2.0, it was compared to scores on the CD-RISC. Resilience refers to the ability to adapt successfully despite risks and negative consequences [46]. Resilience serves as a protective factor against the development of drug or behavioral addiction problems [47]. Both human studies and animal models suggest that individuals with strong resilience exhibit decreased tendencies for seeking highly palatable substances and reduced compulsivity [48]. Animal models indicate that the neurobiological pathways associated with resilience and susceptibility to FA-like behaviors are influenced by enhanced synaptic glutamatergic transmission in the medial prefrontal cortex (mPFC) and nucleus accumbens, which are modulated by the endocannabinoid and dopaminergic signaling systems [49]. The present study replicated previous findings by demonstrating a negative association between resilience and FA [18].

Several limitations should be acknowledged in this study. Firstly, data collection was conducted through an online survey. While online surveys offer advantages such as cost-effectiveness and efficiency in data gathering, they may introduce limitations in terms of representativeness and potential biases. Secondly, the gender ratio in the current sample was unbalanced. Future research would benefit from a more gender-balanced sample to enhance the generalizability of the findings. Furthermore, it is recommended that the psychometric properties of mPYFAS 2.0 be reevaluated in clinical populations in future studies. This would provide valuable insights into the tool's applicability and validity in diagnosing FA in clinical settings.

## Conclusion

In conclusion, this study has successfully shown that the Persian translation of mYFAS 2.0 exhibits strong psychometric characteristics, closely resembling those of the original version. The high internal consistency of the Persian mYFAS 2.0 establishes its usefulness as an effective instrument for investigating food addiction in the Persian-speaking population.

### Supplementary Information

**Additional file 1. **Questionnaires.

## Data Availability

The raw data supporting the conclusions of this article will be sent and available by the corresponding author upon request.
