# Supplementary material for: Confirmatory factor analysis and gender invariance of Persian version of the modified Yale food addiction scale (mPYFAS) 2.0: insight from a large scale Iranian sample
Source: J Eat Disord. 2024 Jan 23;12:14. doi: 10.1186/s40337-023-00962-1 (PMC10804513; doi:10.1186/s40337-023-00962-1)
Supplement: Supplementary file 1 — Additional file 1. Questionnaires. [file 40337_2023_962_MOESM1_ESM.docx]

**Modified Yale Food Addiction Scale Version 2.0**

This survey asks about your eating habits in the past year. People sometimes have difficulty controlling how much they eat of certain foods such as:

- Sweets like ice cream, chocolate, doughnuts, cookies, cake, candy
- Starches like white bread, rolls, pasta, and rice
- Salty snacks like chips, pretzels, and crackers
- Fatty foods like steak, bacon, hamburgers, cheeseburgers, pizza, and French fries
- Sugary drinks like soda pop, lemonade, sports drinks, and energy drinks

When the following questions ask about “CERTAIN FOODS” please think of ANY foods or beverages similar to those listed in the food or beverage groups above or ANY OTHER foods you have had difficulty with in the past year

| IN THE PAST 12 MONTHS: | Never | Less than  monthly | Once a month | 2-3  times a month | Once a  week | 2-3  times a week | 4-6  times a week | Every Day |
| --- | --- | --- | --- | --- | --- | --- | --- | --- |
| 1.I ate to the point where I felt physically ill | 0 | 1 | 2 | 3 | 4 | 5 | 6 | 7 |
| 2.I spent a lot of time feeling sluggish or tired from overeating. | 0 | 1 | 2 | 3 | 4 | 5 | 6 | 7 |
| 3.I avoided work, school or social activities because I was afraid I would overeat there. | 0 | 1 | 2 | 3 | 4 | 5 | 6 | 7 |
| 4.If I had emotional problems because I hadn’t eaten certain foods, I would eat those foods to feel better. | 0 | 1 | 2 | 3 | 4 | 5 | 6 | 7 |
| 5.My eating behavior caused me a lot of distress. | 0 | 1 | 2 | 3 | 4 | 5 | 6 | 7 |
| 6.I had significant problems in my life because of food and eating. These may have been problems with my daily routine, work, school, friends, family, or health. | 0 | 1 | 2 | 3 | 4 | 5 | 6 | 7 |
| 7.My overeating got in the way of me taking care of my family or doing household chores. | 0 | 1 | 2 | 3 | 4 | 5 | 6 | 7 |
| 8.I kept eating in the same way even though my eating caused emotional problems. | 0 | 1 | 2 | 3 | 4 | 5 | 6 | 7 |
| 9.Eating the same amount of food did not give me as much enjoyment as it used to. | 0 | 1 | 2 | 3 | 4 | 5 | 6 | 7 |
| 10.I had such strong urges to eat certain foods that I couldn’t think of anything else. | 0 | 1 | 2 | 3 | 4 | 5 | 6 | 7 |
| 11.I tried and failed to cut down on or stop eating certain foods. | 0 | 1 | 2 | 3 | 4 | 5 | 6 | 7 |
| 12.I was so distracted by eating that I could have been hurt (e.g., when driving a car, crossing the street, operating machinery). | 0 | 1 | 2 | 3 | 4 | 5 | 6 | 7 |
| 13.My friends or family were worried about how much I overate. | 0 | 1 | 2 | 3 | 4 | 5 | 6 | 7 |

**The BES is a 16-item questionnaire**

The BES is a 16-item questionnaire assessing the presence of certain binge eating behaviors which may be indicative of an eating disorder.

Below are groups of statements about behavior, thoughts, and emotional states. Please indicate which statement in each group **best describes how you feel**.

Top of Form

- - I don’t feel self-conscious about my weight or body size when I’m with others.
  - I feel concerned about how I look to others, but it normally does not make me feel disappointed with myself.
  - I do get self-conscious about my appearance and weight which makes me feel disappointed in myself.
  - I feel very self-conscious about my weight and frequently, I feel intense shame and disgust for myself. I try to avoid social contacts because of my self-consciousness.
  - I don’t have any difficulty eating slowly in the proper manner.
  - Although I seem to “gobble down” foods, I don’t end up feeling stuffed because of eating too much.
  - At times, I tend to eat quickly and then, I feel uncomfortably full afterwards.
  - I have the habit of bolting down my food, without really chewing it. When this happens I usually feel uncomfortably stuffed because I’ve eaten too much.
  - I feel capable to control my eating urges when I want to.
  - I feel like I have failed to control my eating more than the average person.
  - I feel utterly helpless when it comes to feeling in control of my eating urges.
  - Because I feel so helpless about controlling my eating I have become very desperate about trying to get in control.
  - I don’t have the habit of eating when I’m bored.
  - I sometimes eat when I’m bored, but often I’m able to “get busy” and get my mind off food.
  - I have a regular habit of eating when I’m bored, but occasionally, I can use some other activity to get my mind off eating.
  - I have a strong habit of eating when I’m bored. Nothing seems to help me break the habit.
  - I’m usually physically hungry when I eat something.
  - Occasionally, I eat something on impulse even though I really am not hungry.
  - I have the regular habit of eating foods, that I might not really enjoy, to satisfy a hungry feeling even though physically, I don’t need the food.
  - Although I’m not physically hungry, I get a hungry feeling in my mouth that only seems to be satisfied when I eat a food, like a sandwich, that fills my mouth. Sometimes, when I eat the food to satisfy my mouth hunger, I then spit the food out so I won’t gain weight.
  - I don’t feel any guilt or self-hate after I overeat.
  - After I overeat, occasionally I feel guilt or self-hate.
  - Almost all the time I experience strong guilt or self-hate after I overeat.
  - I don’t lose total control of my eating when dieting even after periods when I overeat.
  - Sometimes when I eat a “forbidden food” on a diet, I feel like I “blew it” and eat even more.
  - Frequently, I have the habit of saying to myself, “I’ve blown it now, why not go all the way” when I overeat on a diet. When that happens I eat even more.
  - I have a regular habit of starting strict diets for myself, but I break the diets by going on an eating binge. My life seems to be either a “feast” or “famine.”
  - I rarely eat so much food that I feel uncomfortably stuffed afterwards.
  - Usually about once a month, I eat such a quantity of food, I end up feeling very stuffed.
  - I have regular periods during the month when I eat large amounts of food, either at mealtime or at snacks.
  - I eat so much food that I regularly feel quite uncomfortable after eating and sometimes a bit nauseous.
  - My level of calorie intake does not go up very high or go down very low on a regular basis.
  - Sometimes after I overeat, I will try to reduce my caloric intake to almost nothing to compensate for the excess calories I’ve eaten.
  - I have a regular habit of overeating during the night. It seems that my routine is not to be hungry in the morning but overeat in the evening.
  - In my adult years, I have had week-long periods where I practically starve myself. This follows periods when I overeat. It seems I live a life of either “feast or famine.”
  - I usually am able to stop eating when I want to. I know when “enough is enough.”
  - Every so often, I experience a compulsion to eat which I can’t seem to control.
  - Frequently, I experience strong urges to eat which I seem unable to control, but at other times I can control my eating urges.
  - I feel incapable of controlling urges to eat. I have a fear of not being able to stop eating voluntarily.
  - I don’t have any problem stopping eating when I feel full.
  - I usually can stop eating when I feel full but occasionally overeat leaving me feeling uncomfortably stuffed.
  - I have a problem stopping eating once I start and usually I feel uncomfortably stuffed after I eat a meal.
  - Because I have a problem not being able to stop eating when I want, I sometimes have to induce vomiting to relieve my stuffed feeling.
  - I seem to eat just as much when I’m with others (family, social gatherings) as when I’m by myself.
  - Sometimes, when I’m with other persons, I don’t eat as much as I want to eat because I’m self-conscious about my eating.
  - Frequently, I eat only a small amount of food when others are present, because I’m very embarrassed about my eating.
  - I feel so ashamed about overeating that I pick times to overeat when I know no one will see me. I feel like a “closet eater.”
  - I eat three meals a day with only an occasional between meal snack.
  - I eat 3 meals a day, but I also normally snack between meals.
  - When I am snacking heavily, I get in the habit of skipping regular meals.
  - There are regular periods when I seem to be continually eating, with no planned meals.
  - I don’t think much about trying to control unwanted eating urges.
  - At least some of the time, I feel my thoughts are pre-occupied with trying to control my eating urges.
  - I feel that frequently I spend much time thinking about how much I ate or about trying not to eat anymore.
  - It seems to me that most of my waking hours are pre-occupied by thoughts about eating or not eating. I feel like I’m constantly struggling not to eat.
  - I don’t think about food a great deal.
  - I have strong cravings for food but they last only for brief periods of time.
  - I have days when I can’t seem to think about anything else but food.
  - Most of my days seem to be pre-occupied with thoughts about food. I feel like I live to eat.
  - I usually know whether or not I’m physically hungry. I take the right portion of food to satisfy me.
  - Occasionally, I feel uncertain about knowing whether or not I’m physically hungry. At these times it’s hard to know how much food I should take to satisfy me.
  - Even though I might know how many calories I should eat, I don’t have any idea what is a “normal” amount of food for me.

Bottom of Form

**Barratt Impulsiveness Scale 11 (BIS-11)**

Directions: People differ in the ways they act and think in different situations. This is a test to measure some of the ways in which you act and think. Read each statement and put an X on the appropriate circle on the right side of this page. Do not spend too much time on any statement. Answer quickly and honestly **Rarely/Never Occasionally Often Almost Always/Always**

1. I plan tasks carefully.
2. I do things without thinking
3. I make-up my mind quickly.
4. I am happy-go-lucky.
5. I don’t ‘‘pay attention.”
6. I plan trips well ahead of time.
7. I am self controlled.
8. I concentrate easily.
9. I save regularly.
10. I ‘‘squirm” at plays or lectures.
11. I am a careful thinker.
12. I plan for job security.
13. I say things without thinking.
14. I like to think about complex problems.
15. I change jobs.
16. I get easily bored when solving thought problems.
17. I act on the spur of the moment.
18. I change residences.
19. I buy things on impulse.
20. I change hobbies.
21. I spend or charge more than I earn.
22. I often have extraneous thoughts when thinking.
23. I am more interested in the present than the future.
24. I am restless at the theater or lectures.
25. I am future oriented.

**The Connor-Davidson Resilience Scale (CD-RISC-25)**

0 – Not true at all.
1 – Rarely true.
2 – Sometimes true.
3 – Often true.
4 – True nearly all the time.

1. I am able to adapt when changes occur.
2. I have one close and secure relationship.
3. Sometimes fate or God helps me.
4. I can deal with whatever comes my way.
5. Past successes give me confidence.
6. I try to see the humorous side of things when I am faced with problems.
7. Having to cope with stress can make me stronger.
8. I tend to bounce back after illness, injury or other hardships.
9. I believe most things happen for a reason.
10. I make my best effort, no matter what.
11. I believe I can achieve my goals, even if there are obstacles.
12. Even when hopeless, I do not give up.
13. In times of stress, I know where to find help.
14. Under pressure, I stay focused and think clearly.
15. I prefer to take the lead in problem-solving.
16. I am not easily discouraged by failure.
17. I think of myself as a strong person when dealing with life’s challenges and difficulties.
18. I make unpopular or difficult decisions.
19. I am able to handle unpleasant or painful feelings like sadness, fear, and anger.
20. I have to act on a hunch.
21. I have a strong sense of purpose in life.
22. I feel like I am in control.
23. I like challenges.
24. I work to attain goals.
25. I take pride in my achievements.
